# MLVA and MLST typing of *Brucella* from Qinghai, China

**DOI:** 10.1186/s40249-016-0123-z

**Published:** 2016-04-13

**Authors:** Jun-Ying Ma, Hu Wang, Xue-Fei Zhang, Li-Qing Xu, Gui-Ying Hu, Hai Jiang, Fang Zhao, Hong-Yan Zhao, Dong-Ri Piao, Yu-Min Qin, Bu-Yun Cui, Gong-Hua Lin

**Affiliations:** Qinghai Institute for Endemic Disease Prevention and Control, Xining, 811602 Qinghai China; State Key Laboratory for Infectious Disease Prevention and Control, Collaborative Innovation Center for Diagnosis and Treatment of Infectious Diseases, National Institute for Communicable Disease Control and Prevention, Chinese Center for Disease Control and Prevention, Beijing, 102206 China; Key Laboratory of Adaptation and Evolution of Plateau Biota, Northwest Institute of Plateau Biology, Chinese Academy of Sciences, Xining, 810008 Qinghai China

**Keywords:** *Brucella*, Molecular identification, Genotype, Zoonotic host

## Abstract

**Background:**

The Qinghai-Tibet Plateau (QTP) of China is an extensive pastoral and semi-pastoral area, and because of poverty and bad hygiene conditions, *Brucella* is highly prevalent in this region. In order to adequately prevent this disease in the QTP region it is important to determine the identity of *Brucella* species that caused the infection.

**Methods:**

A total of 65 *Brucella* isolates were obtained from human, livestock and wild animals in Qinghai, a Chinese province in east of the QTP. Two molecular typing methods, MLVA (multi-locus variable-number tandem-repeat analysis) and MLST (multi locus sequence typing) were used to identify the species and genotypes of these isolates.

**Findings:**

Both MLVA and MLST typing methods classified the 65 isolates into three species, *B. melitensis*, *B. abortus* and *B. suis*, which included 60, 4 and 1 isolates respectively. The MLVA method uniquely detected 34 (Bm01 ~ Bm34), 3 (Ba01 ~ Ba03), and 1 (Bs01) MLVA-16 genotypes for *B. melitensis*, *B. abortus* and *B. suis*, respectively. However, none of these genotypes exactly matched any of the genotypes in the Brucella2012 MLVA database. The MLST method identified five known ST types: ST7 and ST8 (*B. melitensis*), ST2 and ST5 (*B. abortus*), and ST14 (*B. suis*). We also detected a strain with a mutant type (3-2-3-2-?-5-3-8-2) of ST8 (3-2-3-2-1-5-3-8-2). Extensive genotype-sharing events could be observed among isolates from different host species.

**Conclusions:**

There were at least three *Brucella* (*B. melitensis*, *B. abortus* and *B. suis*) species in Qinghai, of which *B. melitensis* was the predominant species in the area examined. The *Brucella* population in Qinghai was very different from other regions of the world, possibly owing to the unique geographical characteristics such as extremely high altitude in QTP. There were extensive genotype-sharing events between isolates obtained from humans and other animals. Yaks, sheep and blue sheep were important zoonotic reservoirs of brucellosis causing species found in humans.

**Electronic supplementary material:**

The online version of this article (doi:10.1186/s40249-016-0123-z) contains supplementary material, which is available to authorized users.

## Multilingual abstracts

Please see Additional file 1 for translations of the abstract into the six official working languages of the United Nations.

## Introduction

Brucellosis is one of the most widespread and severe zoonotic diseases across the world. This disease affects mammals and is transmitted to humans by animals through direct contact with contaminated animal fluids or indirectly through consumption of unpasteurized milk products [1]. Brucellosis in domestic mammals and humans is the cause of huge economic burden and medical challenges globally, especially in poor regions [2–5]. The causal agent of brucellosis, *Brucella* genus, currently contains 10 species: *B. abortus*, *B. suis*, *B. melitensis*, *B. ovis*, *B. canis*, *B. neotomae*, *B. ceti*, *B. pinnipedialis*, *B. microti*, and *B. inopinata*. Some species are further subdivided into biovars, i.e., *B. melitensis* bv.1 to 3, *B. abortus* bv. 1 to 6 and 9, and *B. suis* bv. 1 to 5 [6]. Most species with a preferential host and three other species (*B. abortus*, *B. melitensis*, and *B. suis*) remain the principal causes of zoonotic potential [1].

Since different species or biovars within a species of *Brucella* have different epidemiological significances, in order to effectively prevent this disease, it is important to identify the species or strain of *Brucella* that causes infection. Most previous molecular subtyping tools and “classical biotyping” methods lack sufficient discriminatory power for epidemiological investigations. Recently, two molecular typing methods called MLVA (multi-locus variable-number tandem-repeat analysis) and MLST (multi locus sequence typing) have emerged as useful tools for identifying and genotyping *Brucella* isolates [3, 7–9].

**Fig. 1:**
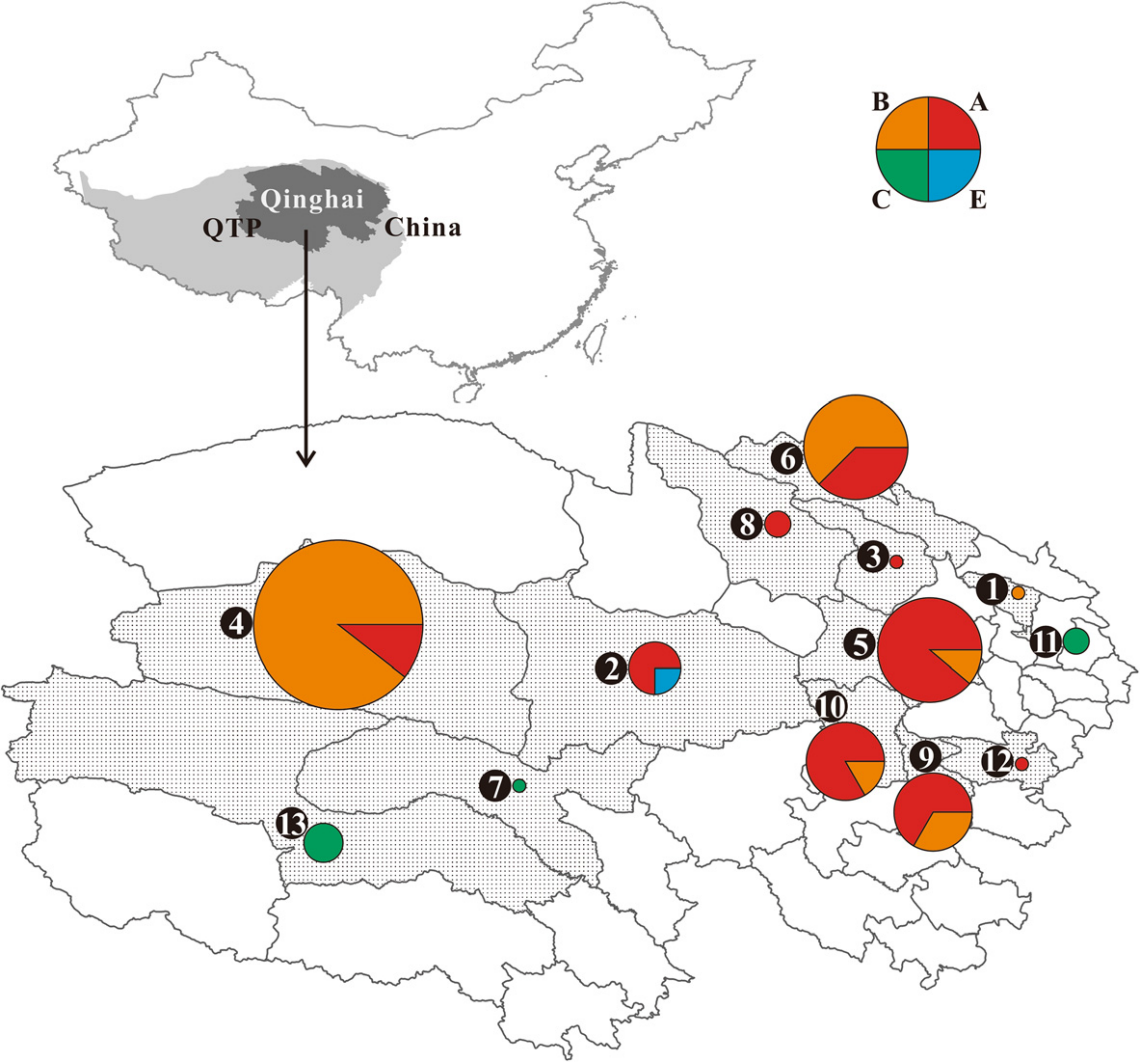
Geographic distribution of *Brucella* samples in Qinghai, China. QTP, Qinghai-Tibet Plateau; the dotted counties (NO. 1 ~ 13) correspond to the 13 counties (Locality ID 1 ~ 13) in Table 1. The pie charts showed the composition (the relative pie size corresponds to the number of strains) of the four branches (A, B, C, and E) of *B. melitensis*

The Qinghai-Tibetan Plateau (QTP) is the largest and highest plateau on Earth, with an area of 2.5 × 10^6^ km^2^ and an average elevation of 4,000 m above sea level [10]. The QTP is an extensive pastoral and semi-pastoral area, and owing to poverty and bad hygiene, *Brucella* is highly endemic to this region among yaks (*Bos grunniens*) [11, 12] and humans [13]. It should be mentioned that, probably due to technical difficulties, most previous studies on *Brucella* in the QTP merely examined infection surveillance, without paying adequate attention to the study of taxonomic identification of *Brucella* species. In the present study we collected a considerable number of *Brucella* samples from Qinghai Province, (Table 1) which is located in the northeastern corner of the QTP and constitutes >25 % of the area of the plateau (Fig. 1). We used both MLVA as well as MLST methods to type these samples and determine the composition characteristics of *Brucella* in the plateau area.

**Table 1:** Geographic distribution of *Brucella* samples in Qinghai, China

Locality ID	County	Sample ID	Sample size
1	Datong	Ma10, Ma50	2
2	Dulan	Ma37, Ma51, Ma52, Ma53, Ma54, Ma55, Ma56, Ma57, Ma58	9
3	Gangcha	Ma17	1
4	Golmud	Ma13, Ma16, Ma18, Ma19, Ma20, Ma21, Ma26, Ma27, Ma28, Ma29, Ma31, Ma32, Ma33	13
5	Gonghe	Ma03, Ma12, Ma22, Ma24, Ma25, Ma30, Ma34, Ma35	8
6	Qilian	Ma39, Ma40, Ma41, Ma42, Ma43, Ma44, Ma45, Ma46	8
7	Qumalai	Ma23	1
8	Tianjun	Ma04, Ma38	2
9	Tongde	Ma36, Ma59, Ma60, Ma61, Ma62, Ma63, Ma64	7
10	Xinghai	Ma01, Ma02, Ma05, Ma06, Ma08, Ma09, Ma15	7
11	Xining	Ma07, Ma14, Ma65	3
12	Zeku	Ma11	1
13	Zhiduo	Ma47, Ma48, Ma49	3

## Methods

### Ethics statement

This study is a retrospective investigation of our historical collection with modern typing methods. Therefore, the study does not involve the collection or reporting of patient data. No animal work was carried out with the obtained results.

### Bacterial strains and DNA preparation

A total of 65 isolates were examined: 31 recovered from human, 15 from sheep (*Ovis aries*), 11 from blue sheep (*Pseudois nayaur*), 6 from yaks (*Bos mutus grunniens*), and 1 each from cattle (*Bos primigenius taurus*) and Tibetan gazelle (*Procapra picticaudata*). Bacterial strains were cultured on Trypticase soy agar containing 5 % sheep blood (BD Diagnostic Systems, China Ltd., China) at 37 °C for 48 h. Total genomic DNA was extracted using the DNeasy Blood & Tissue Kit (Qiagen, Germany) as per the manufacturer’s recommended protocol.

### MLVA genotyping

MLVA was performed as described earlier [9]. Briefly, 16 primer pairs were designed and classified as belonging to one of the three panels: panel 1 (bruce06, bruce08, bruce11, bruce12, bruce42, bruce43, bruce45, and bruce55), panel 2A (bruce18, bruce19, and bruce21), and panel 2B (bruce04, bruce07, bruce09, bruce16, and bruce30). PCR amplifications were performed in 40 μL reaction volumes, 5 μL of these were loaded in to 2 % (panel 1) or 3 % (panels 2A and 2B) agarose gels containing 0.5 μg/ml of ethidium bromide, visualized under UV light, and photographed. Band intensities were estimated using BioNumerics version 5.1 (Applied Maths, Belgium) and were then converted to repeat units by following the published allele numbering system [7].

In order to improve the genotyping accuracy, PCR products were also directly sequenced after purification. The sequences were aligned and the numbers of repeat units were checked in MEGA version 5 [14] using *B. melitensis* 16 M genome sequence as a reference (GenBank accession number NC_003317.1 and NC_003318.1). Clustering analysis was based on the categorical coefficient and unweighted pair group method using arithmetic averages (UPGMA) method provided in BioNumerics. The resulting genotypes were compared using the web-based Brucella2012 MLVA database (http://mlva.u-psud.fr/).

### MLST genotyping

Nine distinct genomic loci, including seven housekeeping genes (*gap*, *aroA*, *glk*, *dnaK*, *gyrB*, *trpE*, and *cobQ*), one outer membrane protein (*omp25*), and one intergenic fragment (int-*hyp*), were selected for MLST genotyping [15]. Similar to the MLVA process described, PCR amplifications were performed in 40 μL reaction volumes using primer sequences as previously described [3]. Sequences obtained from purified PCR products were aligned using MEGA program and verified by visualizing. MLST sequences (GenBank accession number AM694191 ~ AM695630) of the strains described by Whatmore et al. [3] were downloaded and a local BLAST database was built using makeblastdb program of BLAST+ program package version 2.2.31 [16]. The sequences were matched with the local database using blastn program of the BLAST+ program package.

The BLAST results were parsed using BLAST Parser program version 1.2.6 [17]. Distinct alleles identified at the nine selected loci were each given a numerical designation according to sequence of defined alleles. If the sequence was different from those defined previously, it was designated as a new allele. Each sequence type over all loci (ST) was predicted using web-based MLST service (BrucellaBase, http://59.99.226.203/brucellabase/mlst.html). Phylogenetic relationships of combined sequences were inferred using the Neighbor-Joining method in BrucellaBase.

We could not identify biovars within strains, because it was difficult to distinguish among them using either MLVA16 or MLST markers [3, 18, 19].

## Findings

### MLVA results

**Fig. 2:**
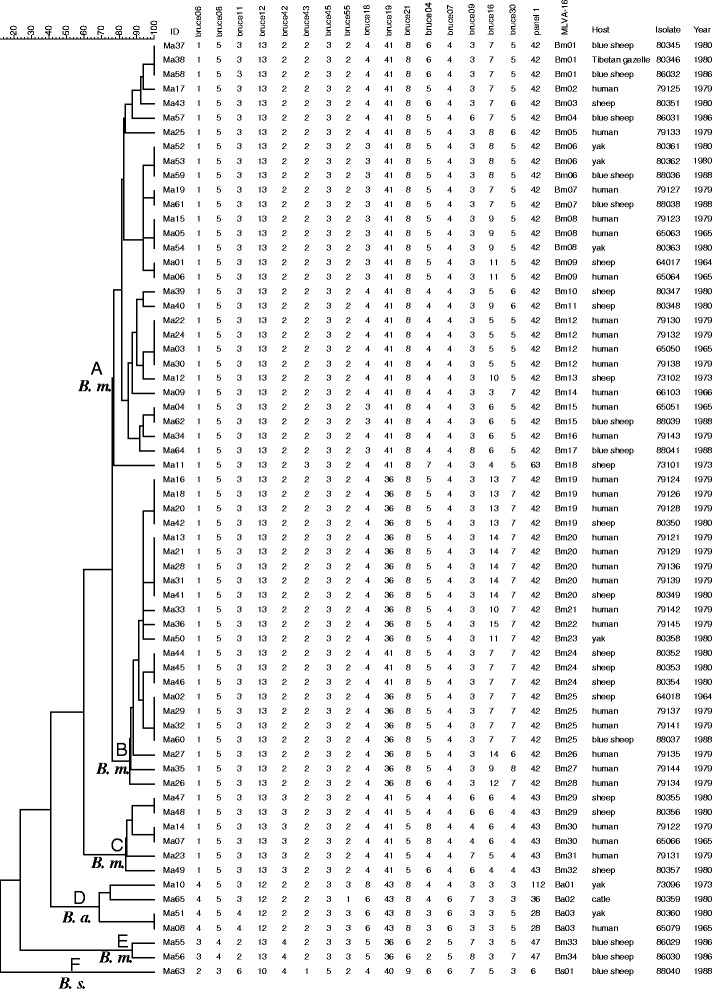
Dendrogram based on the MLVA genotyping assay showing relationships of the 65 *Brucella* isolates. ID: serial number for the 65 isolates; MLVA type: panel 1 and MLVA-16 genotypes; Host: the hosts from which the bacteria was isolated; Isolate: isolate name in the laboratory in which the DNA extraction was done; Year: the years when the strains were collected

Using panel 1 markers, the present population clustered into eight known genotypes: 42 (1-5-3-13-2-2-3-2; *N* = 51), 43 (1-5-3-13-3-2-3-2; *N* = 6), 47 (3-4-2-13-4-2-3-3; *N* = 2), 28 (4-5-4-12-2-2-3-3; *N* = 2), 63 (1-5-3-13-2-3-3-2; *N* = 1), 36 (4-5-3-12-2-2-3-1; *N* = 1), 112 (4-5-3-12-2-2-3-3; *N* = 1), and 6 (2-3-6-10-4-1-5-2; *N* = 1). The Clustering analysis showed that the 65 isolates formed six main clusters (A ~ F). Cluster A had two genotypes (42 and 63); cluster B, C, E and F had a single genotype 42, 43, 47 and 6, respectively; cluster D had three genotypes (112, 36, and 28) (Fig. 2). According to Brucella2012 MLVA database and based on panel 1 markers we identified our samples as containing three species: *B. melitensis* (genotype 42, 43 and 47; cluster A, B, C and E), *B. abortus* (genotype 28, 36 and 112; cluster D) and *B. suis* (genotype 6; cluster F).

Based on a previous study [20], the *B. melitensis* strains were sub-grouped into “East Mediterranean” group (genotype 42 and 43) and “American” group (genotype 47). Considering all three MLVA panels, 34 (Bm01 ~ Bm34), 3 (Ba01 ~ Ba03), and 1 (Bs01) MLVA-16 genotypes were identified for *B. melitensis*, *B. abortus* and *B. suis* isolates respectively (Fig. 2). No genotypes completely matched any of the genotypes in the Brucella2012 MLVA database.

### MLST results

A total of five known MLST genotypes were identified: ST7 (3-5-3-2-1-5-2-10-2; *N* = 2), ST8 (3-2-3-2-1-5-3-8-2; *N* = 57), ST2 (2-1-2-2-1-3-1-1-1; *N* = 2), ST5 (2-1-1-2-1-4-1-1-1; *N* = 2), ST14 (1-6-4-1-4-3-5-2-1; *N* = 1). The Neighbor-Joining clustering showed that the 65 isolates formed four main clusters – a, d, e and f. Of these, clusters a, e and f correspond to genotypes ST8, ST7 and ST14 respectively while cluster d corresponds to two genotypes, ST2 and ST5. According to Whatmore et al. [3], genotypes ST7 and ST8 belong to *B. melitensis*, ST2 and ST5 belong to *B. abortus*, and ST14 belongs to *B. suis*.

The *gyrB* sequence of strain Ma06 was different from any of known alleles (allele 1 ~ 6). Its sequence closely matched that of allele 1 (identical except for a di-nucleotide change of GC to AA on site 405 ~ 406 relative to the *gyrB* allele 1 sequence or on 2504 ~ 2505 sites relative to the ST8 sequence). Considering all MLST alleles, the strain Ma06 could be viewed as a mutant variant (3-2-3-2-?-5-3-8-2) of ST8 (3-2-3-2-1-5-3-8-2).

## Discussion

**Fig. 3:**
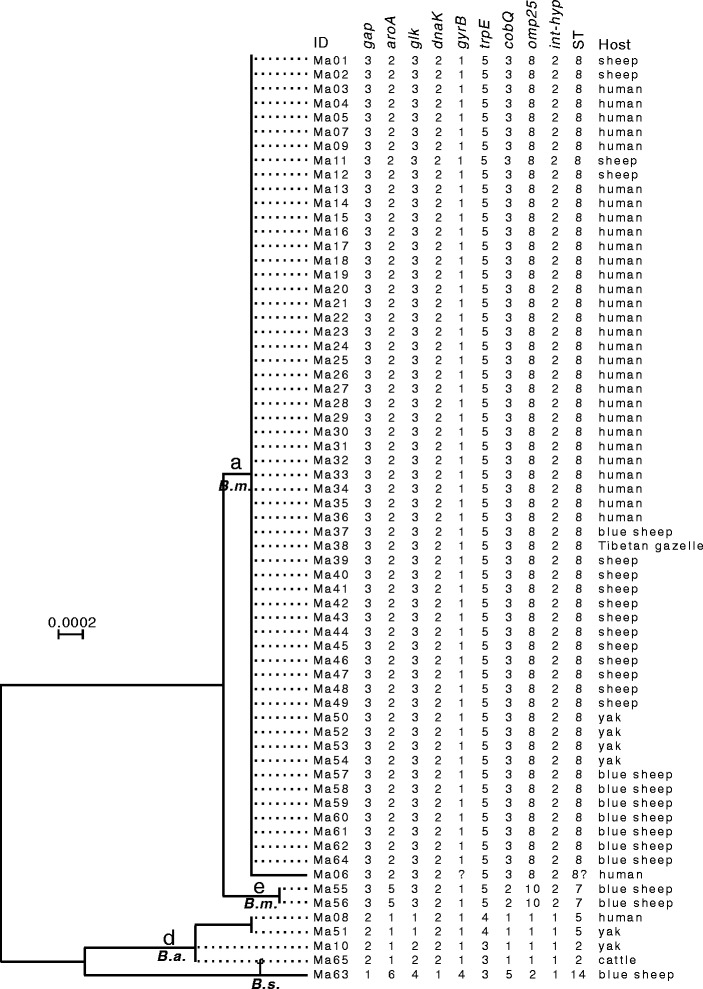
Dendrogram based on the MLST genotyping assay showing relationships of the 65 *Brucella* isolates. ID: serial number for the 65 isolates; ST: MLST genotypes; Host: the hosts from which the bacteria was isolated. (Also see Fig. 2 for additional information)

In this study we used both MLVA and MLST methods to identify *Brucella* species in the east of the Qinghai-Tibet Plateau. Although there were some minor incongruences for e.g., in clustering results (Fig. 2 and Fig. 3), both methods showed a consistent conclusion that there were at least three *Brucella* species in the sample analyzed. Of the 65 isolates 60, 4, and 1 were respectively identified to *B. melitensis*, *B. abortus*, and *B. suis*, indicating that *B. melitensis* was a predominant species on the plateau. Interestingly however, when searching in the Brucella2012 MLVA database none of the genotypes found in our study were identical to any of the genotypes in the database. Based on MLST the ST8 seems to be the main ST type in the QTP region. Moreover, there was also a unique genotype (strain Ma06) which has not been reported anywhere before. These results demonstrated that the *Brucella* population found in the QTP region was very different from that in other regions. We attribute this to relatively isolated and special environment of the plateau. Extremely high altitudes make it difficult for lowland livestock breeds or wild animals to survive, thereby favoring only the endemic ones. Hence, there was a very limited breeds exchange between QTP and other regions, which may have consequently formed a unique local *Brucella* population.

Based on MLVA-16, ten genotypes were shared by two or more host species. To elaborate further, four genotypes (Bm09, Bm19, Bm20, and Bm25) were shared between humans and sheep, and two (Bm08 and Ba03) were shared between humans and yaks (Fig. 2). Based on MLST, the ST8 type was also shared between humans and four other animals (sheep, yaks, blue sheep and Tibetan gazelle); the ST5 type was shared between humans and yaks (Fig. 3). Sheep and yaks are the main livestock on QTP and it is therefore not surprising that these animals pose a heavy zoonotic disease threat to humans. It should be noted that, all sheep and four out of six yaks were infected by *B. melitensis*, indicating that this bacterial species should be paid special attention to in both host species. It is well known that different bacterial species need different vaccine strains. For instance, there are three vaccine strains M5, S19 and S2, which are used to immunize animals in China for *B. melitensis*, *B. abortus*, and *B. suis*, respectively [21]. Because yaks could be infected by either *B. melitensis* or *B. abortus*, we suggest based on our study that both the *B. melitensis* type (M5) and *B. abortus* type vaccines (S19) should be tried in this animal.

The blue sheep were infected by all three *Brucella* species including *B. suis*, which is generally carried by pigs [1, 22]. More importantly, this animal shared *Brucella* genotypes with human (Bm07, Bm15 and Bm25), yaks (Bm06), and Tibetan gazelle (Bm01), indicating this animal is an important natural repertory for *Brucella*. Since there is obvious spatial and diet overlap between blue sheep and domestic livestock [23], we suggest that this animal is also an important infection source of *Brucella* in humans.

Additionally, due to high genetic diversity of *B. melitensis*, we also discuss the geographic distribution features of the strains. Figure 1 showed that 7 counties (locality ID: 2, 3, 5, 8, 9, 10, and 12) were dominated by A branch strains; 3 counties (locality ID: 1, 4, and 6) were dominated by B branch strains; 3 counties (locality ID: 7, 11, and 13) contained only C branch strains; and the E branch genotypes appeared only in 1 county (locality ID: 2). These results showed that the *B. melitensis* prevalence in the QTP region might have a considerable regional characteristic i.e., there might be genetic variations among different regions.

Our results for the first time elucidated the composition of species and genotypes of *Brucella* samples in the QTP region. We also presented the host- as well as geographic distributions of the species and genotypes. These results may have some implications for the future disease control programmes in QTP. First, since *B. melitensis* was the predominant species in the study area, special attention should be paid on this species in brucellosis control programs such as livestock vaccination. Second, the genotypes of *Brucella* in the QTP region were very different from other regions, but whether the genetic difference may result in different vaccination efficiencies should be elucidated in the further studies. Third, since there were extensive genotype-sharing events between humans and animals, brucellosis surveillance should be regularly executed on both livestock (sheep and yaks) and wild ungulates (blue sheep).

It should be noted that, as mentioned above, none of the genotypes found in our study were identical to any of the genotypes in the Brucella2012 database, in order to prevent over-interpreting our results, we did not compare our strains with those from other studies. This might limit our understanding of genetic relationships between the *Brucella* populations from QTP and from other regions. Moreover, because of imbalance of sample sizes among different counties and among different hosts, in this study, we were not able to do a detail epidemiological investigation. We suggest that the accumulation of more samples will enable us to further elucidate the genetic characteristics of *Brucella* species in QTP.

## Conclusions

Our study drew three main conclusions: (i) there were at least three *Brucella* (*B. melitensis*, *B. abortus* and *B. suis*) species in the east of QTP and *B. melitensis* was the predominant species in the area studied; (ii) the *Brucella* population in the QTP region was very different from other regions probably due to the unique geographical characteristics e.g., extremely high altitude in QTP; and (iii) there were extensive genotype-sharing events between humans and animals; sheep, yaks and blue sheep were important zoonotic hosts of brucellosis to humans in the area studied.

## Additional file

Additional file 1: Multilingual abstracts in the six official working languages of the United Nations. (PDF 350 kb)

## Abbreviations

MLST: multi locus sequence typing
MLVA: multi-locus variable-number tandem-repeat analysis
QTP: Qinghai-Tibet Plateau
