# Supplementary material for: MLVA and MLST typing of Brucella from Qinghai, China
Source: Infect Dis Poverty. 2016 Apr 13;5:26. doi: 10.1186/s40249-016-0123-z (PMC4830052; doi:10.1186/s40249-016-0123-z)
Supplement: Additional file 1: — Multilingual abstracts in the six official working languages of the United Nations. (PDF 350 kb) [file 40249_2016_123_MOESM1_ESM.pdf]

Translation of the abstract into the five official working languages of the United Nations

تصنيف MLVA وMLST للحمى المالطية (البروسيلا) في تشينغهاى، الصين

يون-يينغ ما، هو وانغ، شيويه فيى تشانغ، لى تشينغ شو، تشي يينغ ها، هاي جيانغ، فانغ تشاو، هونغ يان تشاو، دونغ-ري بياو، يو مين تشين، بو يون تسوى، جونغ- هوا لين

الملخص

الخلفية: إن هضبة تشينغهاى- التبت (QTP) الصينية هي منطقة رعوية وشبه رعوية واسعة، وبسبب الفقر والظروف الصحية السيئة، فالحمى المالطية منتشرة بشكل كبير في هذه المنطقة. وللوقاية من هذا المرض على نحو كافٍ في منطقة تشينغهاي- التبت فمن المهم تحديد هوية أنواع البروسيلا التي تسبب العدوى.

الطرق: تم الحصول على إجمالي 65 عزلات بروسيلا من الإنسان والثروة الحيوانية والحيوانات البرية فى تشينغهاى، المقاطعة الصينية في شرق هضبة تشينغهاي- التبت. استخدمت طريقتين في التصنيف الجزيئي، MLVA (تحليل متعدد لعدد متغير الموضع تكراري جنباً إلى جنب) وMLST (تصنيف متعدد التسلسل الموضعي) لتحديد الأنواع والأنماط الجينية لهذه العزلات.

النتائج: صنفت كلا الطريقتين MLVA وMLST الـ 65 عزلات إلى ثلاثة أنواع، ب. المالطية، و ب. المجهضة و ب. الخنزيرية، والتي شملت 60 و 4 و 1 عزلة على التوالي. كشفت طريقة MLVA بصورة فريدة 34 (Bm01 ~ Bm34) و 3 (Ba01 ~ Ba03)، و 1 (BS01) المورثات MLVA 16 لـ ب. المالطية، و ب. المجهضة و ب. الخنزيرية، على التوالي. ومع ذلك، فإن أياً من هذه المورثات لم يطابق بالضبط أي من التراكيب الوراثية في قاعدة بيانات Brucella2012 MLVA. وقد حددت طريقة MLST خمسة أنواع ST معروفة: ST7 وST8 (ب. المالطية)، ST2 وST5 (ب. المجهضة)، وST14 (ب. الخنزيرية). وقد اكتشفنا أيضاً سلالة ذات نوع متحول (3-2-3-2 -؟ - 5-3-8-2) من ST8 (2-8-3-5-1-2-3-2-3). ويمكن ملاحظة نتائج تقاسم وراثي واسعة بين العزلات من أنواع مضيفة مختلفة.

الاستنتاجات: كان هناك على الأقل ثلاثة أنواع بروسيلا (ب. المالطية، و ب. المجهضة و ب. الخنزيرية) فى تشينغهاى، والتي كان منها ب. المالطية هو النوع السائد في المنطقة التي تم فحصها. كان عدد تجمعات البروسيلا فى تشينغهاى يختلف كثيراً عن مناطق أخرى من العالم، ربما بسبب الخصائص الجغرافية الفريدة مثل العلو الشاهق في هضبة تشينغهاي- التبت. كانت هناك نتائج تقاسم وراثي واسعة بين العزلات التي تم الحصول عليها من البشر والحيوانات الأخرى. وكانت الثيران والأغنام والخراف الزرقاء خزانات حُيَيْوينيّة مهمة للبروسيلا مسببة أنواعاً توجد في البشر.

Translated from English version into Arabic by Free bird, through

TRANSLATORS
WITHOUT BORDERS

青海省布鲁氏菌 MLVA 和 MLST 分型

马俊英，王虎，张雪飞，徐立青，胡桂英，姜海，赵芳，赵鸿雁，朴东日，秦豫民，崔步云，林恭华

摘要

引言：青藏高原有广阔的牧区和半牧区，由于贫困和恶劣的卫生条件，布鲁氏菌病在该地区高度流行。鉴定青藏高原地区布鲁氏菌的种类组成，对有效预防布鲁氏菌病具有重要意义。

方法：从青海省（青藏高原东部省份）的人、家畜和野生动物身上采集到 65 份布鲁氏菌菌株。利用 MLVA（多位点可变数目串联重复序列分型）和 MLST（多位点序列分型）两种分子分型手段，对这些菌株进行物种鉴定和遗传分型。

结果：MLVA 和 MLST 两种分型方法都将 65 份菌株分为 3 个布鲁氏菌物种，其中羊种 60 株、牛种 4 株、猪种 1 株。基于 MLVA-16 分型标准，MLVA 方法检测到 34 个羊种基因型（Bm01~Bm34）、3 个牛种基因

型（Ba01~Ba03）和 1 个猪种基因型（Bs01）。然而，所有这些基因型中，没有一个与 Brucella2012 MLVA 数据库中已登记的基因型完全匹配。MLST 方法检测到 5 种已知的 ST 型：ST7 和 ST8（羊种）、ST2 和 ST5（牛种）、ST14（猪种）。我们同时检测到 ST8 型（3-2-3-2-1-5-3-8-2）的一种突变型（3-2-3-2-?-5-3-8-2）。此外，不同宿主之间共享同一种布鲁氏菌基因型的现象非常普遍。

结论：青海省至少有 3 种（羊种、牛种、猪种）布鲁氏菌分布，其中羊种在本研究区域是主要物种。青海省布鲁氏菌种群在遗传上与世界其他地区的菌群差别很大，这可能与青藏高原独特的地理特征如极高的海拔有关。人和其他动物之间共享同一种布鲁氏菌基因型的现象非常普遍。牦牛、藏羊和岩羊是人类布鲁氏菌病的重要储存宿主。

Translated from English version into Chinese by Lin Gong-hua

Typage MLVA et MLST de la *Brucella* dans la région du Qinghai, Chine

Jun-Ying Ma, Hu Wang, Xue-Fei Zhang, Li-Qing Xu, Gui-Ying Hu, Hai Jiang, Fang Zhao, Hong-Yan Zhao, Dong-Ri Piao, Yu-Min Qin, Bu-Yun Cui, Gong-Hua Lin

Résumé

Contexte : la région du Plateau tibétain du Qinghai (QTP) en Chine est un espace étendu pastoral et semi-pastoral où la *Brucella* affiche une prévalence élevée en raison de la pauvreté et des conditions d'hygiène médiocres qui y règnent. Afin de prévenir cette maladie de manière adéquate dans la région QTP, il est important de déterminer l'identité des espèces de *Brucella* à l'origine de l'infection.

Méthodes : au total, 65 isolats de *Brucella* ont été obtenus chez des êtres humains, du bétail et des animaux sauvages du Qinghai, une province chinoise dans l'est du QTP. Deux méthodes de typage moléculaire ont été utilisées pour identifier les espèces et les génotypes de ces isolats : MLVA (analyse de plusieurs locus VNTR) et MLST (typage génomique multilocus).

Observations :les deux méthodes de typage MLVA et MLST ont permis la classification de 65 isolats en trois espèces différentes : *B. melitensis*, *B. abortus* et *B. suis*, comprenant respectivement 60, 4 et 1 isolat. La méthode MLVA a exceptionnellement permis de détecter respectivement 34 (Bm01~Bm34), 3 (Ba01~Ba03) et 1 (Bs01) génotypes MLVA-16 pour les espèces *B. melitensis*, *B. abortus* et *B. suis*. Néanmoins, aucun de ces génotypes ne correspondait exactement à un quelconque génotype de la base de données MLVA 2012 sur la Brucella. La méthode MLST a permis d'identifier cinq types de ST connus : ST7 et ST8 (*B. melitensis*), ST2 et ST5 (*B. abortus*) et ST14 (*B. suis*). Nous avons aussi détecté une souche avec un type mutant (3-2-3-2-?-5-3-8-2) de ST8 (3-2-3-2-1-5-3-8-2). Des événements étendus de partage de génotype ont pu être observés parmi des isolats provenant de différentes espèces hôtes.

Conclusions : au moins trois espèces de *Brucella* (*B. melitensis, B. abortus* et *B. suis*) ont été identifiées au Qinghai, l'espèce *B. melitensis* étant prédominante dans la région examinée. La population de *Brucella* au Qinghai était très différente des autres régions du monde, probablement en raison des caractéristiques géographiques uniques qui y règnent, notamment l'altitude extrême. Des événements étendus de partage de génotype se sont produits entre des isolats recueillis auprès d'êtres humains et d'autres animaux. Les yacks, les moutons et les moutons bleus constituaient d'importants réservoirs zoonotiques de brucellose à l'origine des espèces identifiées chez l'homme.

Translated from English version into French by eric ragu, through

Мультилокусный анализ VNTR-последовательностей и мультилокусное сиквенсное типирование бруцеллы в Цинхае, Китай.

Юн-Йинг Ма, Ру Ванг, Ксю-Фей Жанг, Ли-Чин Ксю, Гуи-Йинг Ху, Хай Джанг, Фанг Джао, Хонг-Ян Жао, Донг-Ри Пяо, Юу-Мин Чин, Бу-Юун Ку, Гонг-Хуа Лин.

Краткое описание ситуации

Общие сведения: Цинхай-Тибетское нагорье в Китае является обширным пастбищным и полупастбищным районом, и вследствие бедности и плохих гигиенических условий бруцелла широко распространена в этом регионе. Для эффективного предоствращения распространения этого заболевания в регионе Цинхай-Тибетского нагорья важно определить идентификационные характеристики видов бруцеллы, которые вызвали заболевание.

Методы: 65 изолятов бруцеллы были взяты у людей, домашнего скота и диких животных в Цинхае, китайской провинции на востоке Цинхай-Тибетского нагорья. Два молекулярных метода типирования, мультилокусный анализ VNTR-последовательностей и мультилокусное сиквенсное типирование использовались для идентификации видов и генотипов этих изолятов.

Результаты исследования: Оба метода - мультилокусный анализ VNTR-последовательностей и мультилокусное сиквенсное типирование - классифицировали 65 изолятов на три вида: *B. melitensis*, *B. abortus* и *B. suis, которые включали 60, 4 и 1 изолятов соответственно.* Метод мультилокусного анализа VNTR-последовательностей обнаружил 34 (Bm01~Bm34), 3 (Ba01~Ba03), и 1 (Bs01) MLVA-16 генотипа *B. melitensis*, *B. abortus* и *B. suis*, соответственно. Однако, ни один из этих генотипов не соответствовал полностью какому-либо генотипу в базе данных мультилокусного анализа VNTR-последовательностей Бруцелла 2012. Метод мультилокусного сиквенсного типирования выявил пять известных ST-типов: ST7 и ST8 (*B. melitensis*), ST2 и ST5 (*B. abortus*), и ST14 (*B. suis*). Мы также обнаружили штамм с мутированноым типом (3-2-3-2-?-5-3-8-2) ST8 (3-2-3-2-1-5-3-8-2). Обширные схожие признаки генотипов наблюдались среди изолятов, взятых у различных особей-носителей.

Выводы: В Цинхае было выявлено по меньшей мере три вида бруцеллы (*B. melitensis*, *B. abortus* и *B. suis*), из которых *B. melitensis была доминирующим видом в районе исследования.* Популяция бруцеллы в Цинхае сильно отличалась от других регионов мира, возможно, вследствие уникальных географических характеристик, таких как высота Цинхай-Тибетского нагорья. Были выявлены обширные схожие признаки генотипов между изолятами, полученными у людей и других животных. Яки, овцы и голубые овцы являлись важными зоонозными резервуарами видов возбудителей бруцеллёза, встречающихся у людей.

Translated from English version into Russian by Tatiana Glazina, through

Tipificación **MLVA y MLST de la *Brucella* en Qinghai, China**

Jun-Ying Ma, Hu Wang, Xue-Fei Zhang, Li-Qing Xu, Gui-Ying Hu, Hai Jiang, Fang Zhao, Hong-Yan Zhao, Dong-Ri Piao, Yu-Min Qin, Bu-Yun Cui, Gong-Hua Lin

Resumen

Antecedentes: La meseta tibetana Qinghai en China es una zona ampliamente rural y semi-rural y debido a la pobreza y a malas condiciones de higiene, la *Brucella* tiene alta prevalencia en la región. Para poder prevenir esta enfermedad de manera adecuada en la meseta tibetana Qinghai es importante determinar la identidad de las especies de *Brucella* que produjeron la infección.

Métodos: Se obtuvieron un total de 65 cepas de Brucella de seres humanos, ganado y animales salvajes en Qinghai, una provincia ubicada al este de la meseta tibetana Qinghai en China. Se utilizaron dos métodos moleculares de clasificación, MLVA (número variable de repeticiones en tándem) y MLST (tipificación multilocus de secuencias) para identificar las especies y genotipos de dichas cepas.

Hallazgos: Ambos métodos de tipificación, el MLVA y el MLST clasificaron las 65 cepas en tres especies, *B. melitensis*, *B. abortus* y *B. suis*, que incluyeron 60, 4 y 1 cepa respectivamente. El método MLVA detectó de manera única 34 (Bm01~Bm34), 3 (Ba01~Ba03), y 1 (Bs01) genotipos MLVA-16 para *B. melitensis*, *B. abortus* y *B. suis*, respectivamente. Sin embargo, ninguno de estos genotipos concordó de manera exacta con los genotipos en la base de datos MLVA Brucella2012. El método MLST identificó cinco tipos conocidos de ST: ST7 y ST8 (*B. melitensis*), ST2 y ST5 (*B. abortus*), y ST14 (*B. suis*). También detectamos una cepa con un tipo mutante (3-2-3-2-?-5-3-8-2) de ST8 (3-2-3-2-1-5-3-8-2). Se pudo observar una gran cantidad de eventos de genotipo compartido entre las cepas de las distintas especies anfitrionas.

Conclusiones: Hubo por lo menos tres especies de *Brucella* (*B. melitensis*, *B. abortus* y *B. suis*) en Qinghai, de las cuales *B. melitensis* fue la especie predominante en el área examinada. La población de *Brucella* en Qinghai fue muy diferente a la de otras regiones del mundo, posiblemente debido a las particulares características geográficas como las altitudes extremas en la meseta tibetana Qinghai. Se produjeron una gran cantidad de eventos de genotipo compartido entre las cepas obtenidas de seres humanos y otros animales. Los yaks, las ovejas y las cabras azules del Himalaya fueron importantes reservorios zoonóticos para la brucelosis que producen las especies encontradas en seres humanos.

Translated from English version into Spanish by Maria Alejandra Aguada, through

TRANSLATORS WITHOUT BORDERS
